# Outcomes of a radiation sparing approach in medulloblastoma by subgroup in young children: an institutional review

**DOI:** 10.1007/s00381-023-05918-z

**Published:** 2023-04-06

**Authors:** Rebecca Ronsley, Joanna Triscott, Joseph Stanek, S. Rod Rassekh, Amy Lum, Sylvia Cheng, Karen Goddard, Dina McConnell, Caron Strahlendorf, Ash Singhal, Jonathan L. Finlay, Stephen Yip, Christopher Dunham, Juliette Hukin

**Affiliations:** 1 Division of Hematology, Oncology & Bone Marrow Transplant, Department of Pediatrics, Seattle Children’s Hospital and the University of Washington, Seattle, WA USA; 2 Department of BioMedical Research, University of Bern, Bern, Switzerland; 3 Division of Hematology, Oncology & Bone Marrow Transplant, Department of Pediatrics, British Columbia Children’s Hospital, 4480 Oak Street B318, Vancouver, BC V6H 3V4 Canada; 4 Department of Pathology, Vancouver General Hospital, Vancouver, Canada; 5 Department of Radiation Oncology, British Columbia Cancer Agency, Vancouver, Canada; 6 Department of Psychology, British Columbia Children’s Hospital, Vancouver, Canada; 7 Division of Pediatric Neurosurgery, Department of Surgery, British Columbia Children’s Hospital, Vancouver, Canada; 8 Departments of Pediatrics and Radiation Oncology, the Ohio State University College of Medicine, Columbus, OH USA; 9 Department of Pathology, British Columbia Children’s Hospital, Vancouver, Canada; 10 Division of Neurology and Division of Hematology Oncology Bone Marrow Transplant, Department of Pediatrics, British Columbia Children’s Hospital, Vancouver, Canada

**Keywords:** Pediatric, Oncology, Neuro-oncology, Medulloblastoma, Radiation, Survivorship

## Abstract

**Objective:**

To describe disease outcomes including overall survival and relapse patterns by subgroup in young pediatric patients treated for medulloblastoma with a radiation-sparing approach.

**Methods:**

Retrospective analysis of clinical outcomes includes treatment, relapse, and salvage therapy and late effects in children treated for medulloblastoma with a radiation-sparing approach at British Columbia Children’s Hospital (BCCH) between 2000 and 2020.

**Results:**

There were 30 patients (median age 2.8 years, 60% male) treated for medulloblastoma with a radiation-sparing approach at BCCH. Subgroups included Sonic Hedgehog (SHH) (*n* = 14), group 3 (*n* = 7), group 4 (*n* = 6), and indeterminate status (*n* = 3). Three- and 5-year event-free survival (EFS) were 49.0% (30.2–65.4%) and 42.0% (24.2–58.9%) and overall survival (OS) 66.0% (95% CI 46.0–80.1%) and 62.5% (95% CI 42.5 and 77.2%), respectively, with a median follow-up of 9.5 years. Relapse occurred in 12/25 patients following a complete response, of whom six (group 4: *n* = 4; group 3: *n* = 1; unknown: *n* = 1) were successfully salvaged with craniospinal axis (CSA) RT and remain alive at a median follow-up of 7 years. Disease/treatment-related morbidity included endocrinopathies (*n* = 8), hearing loss *n* = 16), and neurocognitive abnormalities (*n* = 9).

**Conclusions:**

This radiation sparing treatment approach for young patients with medulloblastoma resulted in a durable cure in most patients with SHH subgroup medulloblastoma. In those patients with groups 3 and 4 medulloblastoma, relapse rates were high; however, most group 4 patients were salvaged with RT.

## Introduction

Brain tumors are among the leading causes of disease-associated death in children and adolescents [1]. While most malignant brain tumors are best treated with radiation therapy (RT) and neoadjuvant chemotherapy, debate exists as to the best treatment approach for young children, given these patients’ increased susceptibility to RT-induced severe morbidity [1, 2].

In medulloblastoma specifically, there has been significant development of many innovative therapeutic strategies including conventional chemotherapy (for example, the CCG9921, SJYC07, CNS9204, and SFOP protocols), use of intrathecal or intra-Ommaya chemotherapy (for example, the HIT-SKK92 protocol), and marrow-ablative chemotherapy with autologous hematopoietic progenitor cell rescue (including the Head Start studies, CCG 99703, and the Children’s Oncology Group [COG] protocol ACNS 0334) as a means to avoid or postpone RT for young children [3–15].

Radiation sparing protocols utilizing marrow-ablative chemotherapy including the Head Start protocols, the COG ACNS 0334 protocol, and CCG 99703 [3, 4, 7] are widely used for this purpose in major pediatric centers across North America; however, many limit this therapy to those under 3 years of age. In a multi-center North American cohort study of infants treated with CCG 99703, among 53 children under age 3 years, event free survival and overall survival were 69.6% and 76.1%, respectively [7].

The Head Start protocols were originally developed to provide high-dose chemotherapy with the patient’s stem cells transfused to rescue the bone marrow in place of local control with RT for patients up to age 10 [4]. Initial outcome data for medulloblastoma using Head Start I and II protocols demonstrated a 5-year EFS and OS of 52% and 70%, respectively, among 21 children less than 3 years of age [4]. More recently, patients treated on the Head Start III protocol had 5-year EFS and OS of 46 ± 5% and 62 ± 5% for all patients, with 90% OS for those tumors with desmoplastic-nodular histology [16].

In parallel with the development of innovative therapeutic regimens that delay or avoid RT, our understanding of medulloblastoma biology has also advanced considerably over the last decade. We now understand that there are 4 molecular subgroups (WNT, SHH, group 3, and group 4) and likely additional sub-subgroups as well [17–19]. While the WNT subgroup carries an excellent prognosis when compared to the other groups, it is extremely rare in very young children. Within infant medulloblastoma, the SHH subgroup is the most common, followed by group 3 and group 4 [5, 8, 10]. In one North-American retrospective cohort treated according to CCG 99703, patients with SHH and group 3 medulloblastoma had a 5-year PFS of 86.2% (± 7.4%) and 49.1% (± 14%), respectively (*p* = 0.03) [7]. The 5-year PFS without RT for group 3 MB within that cohort was 46.4% [7]. Furthermore, further novel targets beyond subgrouping including PLK1 have been identified that may predict risk within young children with medulloblastoma [20]. As our understanding of the interrelationship between molecular subgroup, treatment, and prognosis is relatively new, standard of care treatment for very young children with medulloblastoma has little variation based on the tumor biology.

The British Columbia Children’s Hospital (BCCH) has used RT sparing approaches to treat young children with medulloblastoma for more than two decades. While it is well understood that subgrouping plays a role in prognostication in medulloblastoma, and there is substantial neurocognitive data on the impact of RT sparing approaches on quality of survival outcomes [21], we do not have a clear understanding of survival, relapse patterns, and long-term morbidity with RT sparing approaches in the context of molecular subgroup. The objective of this study was to describe disease outcomes including overall survival and relapse frequency and pattern by subgroup within pediatric patients treated for medulloblastoma with a RT sparing approach in a single institution.

## Methods

### Setting

The BC Children’s Hospital (BCCH), located in Vancouver, Canada, is the sole tertiary care pediatrics hospital servicing the province of British Columbia (BC) and the Yukon Territory. The Oncology division at BCCH provides care for children with new cancer diagnoses from age 0–17 years. This includes all children with a diagnosis of a CNS tumor.

In 1998, BCCH adopted the “Head Start I” regimen for treatment of young children (under age 7 years) with medulloblastoma and subsequently Head Start II and III. All subjects included in this study were treated as per this protocol, which includes 5 induction cycles of chemotherapy followed by 1 consolidation cycle with high-dose chemotherapy and autologous stem cell transplant. Induction chemotherapy (21 day cycles that include cisplatin 3.5 mg/kg IV on day 1, vincristine 0.05 mg/kg on days 1, 8, 15, cyclophosphamide 65 mg/kg IV, and etoposide 4 mg/kg IV on days 2 and 3) was followed by 28-day cycle of high dose/marrow ablative chemotherapy (thiotepa 300gm/m2 IV on days − 5, − 4, and − 3; etoposide 250 mg/m2 IV on days − 5, − 4, and − 3; carboplatin 500 mg/m2 IV on days − 8, − 7, and − 6) and autologous bone marrow or peripheral blood hematopoietic cell transplant [4] with the goal to avoid or delay RT in young children. The total number of planned cycles was determined based on the current Head Start protocol at the time, with Head Start I, II, and III all including 5 induction cycles and one consolidation cycle. In Head Start II, methotrexate (400 mg/kg IV) was added for disseminated disease, and in Head Start III, it was used for all patients in alternating chemotherapy cycles during induction [4, 16, 22].

### Study design

This study was approved by the British Columbia Children’s and Women’s Hospital and University of British Columbia Research Ethics Boards [approval number H20-00145].

This was a retrospective cohort study of pediatric patients treated with RT sparing/delaying approaches for medulloblastoma at a tertiary care pediatric hospital between January 2000 and January 2020. These dates were chosen based on the time of initiation of RT-sparing approaches for infant medulloblastoma at our institution. The end date was chosen to allow at least 1 year of follow-up for all patients prior to data analysis. Patients treated on the current open study, Head Start 4, were excluded. Subjects were identified using the British Columbia Children’s Hospital Division of Oncology database, and all children under the age of 7 with a diagnosis of medulloblastoma and treated without RT upfront (surgery and chemotherapy only) were included in this study.

Patient medical records were reviewed, and data collection included date of diagnosis, patient demographics and past medical history, medulloblastoma pathologic diagnosis and histology, subgrouping and molecular features, treatment received (including number of induction cycles and consolidation cycles), relapse, salvage therapy, and ultimate patient outcome (alive without disease, alive with disease, death), late effects, and time to follow-up.

Various audiologic testing methods were used to assess hearing depending on the patient’s age and developmental status. Tympanometry was reviewed to determine the integrity of the conductive mechanism at the time of testing. Pure tone air conduction thresholds were evaluated at frequencies 0.25, 0.5, 1, 2, 3, 4, 6, and 8 kHz in decibel (dB) hearing level (HL). Pure tone bone conduction thresholds were assessed at frequencies 0.25, 0.5, 1, 2, 3, and 4 kHz to determine the nature of the hearing impairment (i.e., conductive, sensorineural, or mixed). Click and tone-burst auditory brainstem response (ABR), auditory steady-state response, and/or distortion-product otoacoustic emissions (DPOAE) measurements were evaluated on patients who were unable to participate in conventional audiometric testing due to young age, cognitive or developmental delay, or lack of cooperation.

The International Society for Pediatric Oncology (SIOP) criteria were used to grade hearing loss [23].

At BCCH, patients are referred for neuropsychological assessments to evaluate the impact of CNS tumors and therapy on brain development. Following therapy completion, patients being treated for CNS tumors at our institution are referred for evaluations to assess the presence of late effects using standardized measures of intelligence, memory, attention, and executive skills. Assessments are tailored to the clinical needs of the patient, their age, grade in school, and stamina. As neuro-psychological reports were not available in the medical record for all patients, neuro-cognitive abnormalities were collected from the treating oncologist’s clinical documentation.

### Tumor analysis

Immunohistochemistry analysis was performed on all tumors (including BCTN, GAB1, and YAP1). Those tumors where medulloblastoma molecular subgroup had not been previously analyzed for clinical care were retrospectively analyzed via NanoString.

Patient tumors were assigned molecular subtype with methods described previously [18]. RNA was extracted from scrolls of formalin-fixed paraffin-embedded (FFPE) tissue, and quality checked with Agilent 2100 bioanalyzer. Gene expression measurements were performed using 300 ng RNA per sample with a custom NanoString CodeSet including 22 medulloblastoma subtype gene probes using the nCounter system. Procedures recommended by NanoString Technologies were used for sample preparation, hybridization, detection, scanning, and normalization. Molecular subtype was assigned using previously published training set cohorts from BCCH [19]. Predictive analysis for microarrays (PAM) statistical methods were applied using R statistical programming [version 4.1.2].

### Statistical analysis

The cohort of patients were analyzed in full and by medulloblastoma subgroup (Wnt, SHH, group 3, and group 4). Descriptive statistics of all variables were analyzed using mean and standard deviation (SD) for normally distributed variables and median with range for those that were not normally distributed. Overall survival (OS) and event free survival (EFS) were estimated for each medulloblastoma subgroup using the Kaplan–Meier method.

Statistical significance was defined using a *p* value < 0.05. Analysis was completed using SPSS for Windows version 24 (SPSS, Inc., Chicago, IL).

## Results

### Subject characteristics

Between 2000 and 2020, there were 30 pediatric patients with medulloblastoma treated with RT sparing approaches at our institution and were included in this study. Patient demographics are outlined in Table 1.

**Table 1:** Subject demographics

	**Total** *N* = 30
Mean age in years (SD)	2.81 (1.67)
Male	18 (60%)
Ethnicity	
Caucasian	20
Chinese, Korean, or Japanese	6
South-East Asian	4
Histology	
Anaplastic	4
Classical	18
Nodular/desmoplastic	7
Extensive nodular	1
Subgroup	
SHH	14
Group 3	7
Group 4	6
Unknown	3
Metastatic disease*	12
M1	1
M2	6
M3	5
Initial surgery	
GTR	22
STR	5
Biopsy	3

*CSF* cerebrospinal fluid, *GTR* gross total resection, *SD* standard deviation, *STR* subtotal resection

^*^Dufour C, Beaugrand A, Pizer B, Micheli J, Aubelle MS, Fourcade A, Couanet D, Laplanche A, Kalifa C, Grill J. Metastatic Medulloblastoma in Childhood: Chang’s Classification Revisited. Int J Surg Oncol. 2012;2012:245,385. https://doi.org/10.1155/2012/245385

The median age of the study patients at diagnosis was 2.81 years and 60% were male. Two patients had a diagnosis of speech delay, and one subject had a diagnosis of neurofibromatosis type 2 (NF2) prior to the diagnosis of medulloblastoma. Otherwise, there was no prior medical history in any of the study patients.

Tumor histology within this cohort included classical (*n* = 18), nodular/desmoplastic (*n* = 7), anaplastic (*n* = 4), and medulloblastoma with extensive nodularity (MBEN) (*n* = 1). Metastatic disease was noted in 12 patients at initial presentation, of which disseminated disease (Chang staging M3) was present in 5 patients. Two patients with extensive disseminated disease at diagnosis had anaplastic pathology.

Medulloblastoma sub-grouping done in retrospect on stored samples included SHH (*N* = 14), group 3 (*N* = 7), group 4 (*N* = 6), and indeterminate status (*N* = 3). Indeterminate status was due to insufficient tumor sample in all three of these tumors.

Three of the patients with SHH subtype had a TP53 mutation (determined by both IHC and subsequent FISH analysis). Within those patients with group 3, one was MYC amplified, and within those with group 4, two were N-MYC amplified. Germline testing was completed in 6 patients (including all patients whose tumors harbored a TP53 mutation) and no mutations were found.

### Treatment

Twelve (40%) of the subjects presented with metastatic disease. Disseminated disease to craniospinal axis and CSF was seen in six patients (group 3 *n* = 4, group 4 *n* = 1, and unknown subgroup *n* = 2). Initial surgery included a gross total resection (GTR) (*N* = 22), subtotal resection (STR) (*N* = 5), or biopsy (**N** = 3).

Subjects were treated with two (*N* = 1), three (*N* = 7), and five (*N* = 22) induction cycles of chemotherapy and zero (*N* = 3) and one (*N* = 27) cycles of consolidation therapy with high-dose chemotherapy and autologous stem cell transplant. Number of induction cycles were reduced secondary to treatment-related toxicity in all cases. Twelve subjects (40%) received methotrexate as part of induction chemotherapy.

### Clinical outcomes

**Fig. 1:**
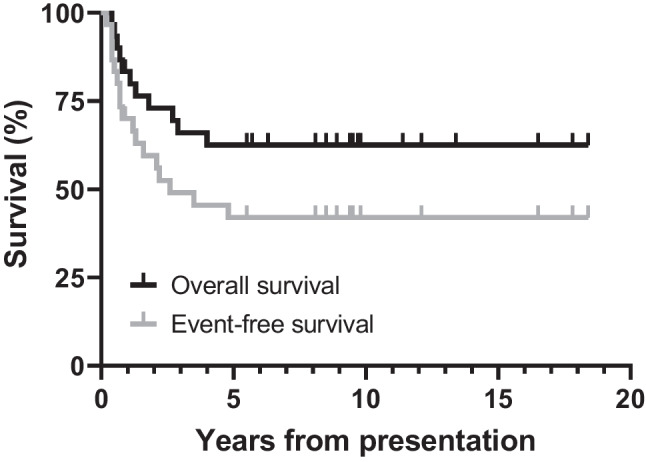
Overall survival and event free survival

Currently, there are 19 patients alive at a mean time to follow-up of 9.5 years. Of these, one patient has a stable nodule, and one patient has a slowly progressive nodule (salvage therapy recommended). Within this cohort of 30 patients, 3-year EFS and 5-year EFS were 49.0% (30.2–65.4%) and 42.0% (24.2–58.9%), respectively (Fig. 1) and 3-year and 5-year OS were 66.0% (95% CI 46.0–80.1%) and 62.5% (42.5–77.2%), respectively.

Response to initial therapy included a complete response (CR) (*N* = 25), partial response (*N* = 3), and progressive disease (*N* = 2). Of those patients who did not have a CR or CCR, none had a GTR upfront and three had disease metastatic to craniospinal axis and CSF at diagnosis. All five of the patients who did not have a CR after initial therapy ultimately died from progressive disease. Three of those patients received methotrexate. Within all 12 patients who received methotrexate as part of induction therapy, 9 (75%) had a complete response to upfront therapy. Of these nine patients, relapse occurred in 4 patients, of which subgroups were group 3 (*N* = 2), group 4 (*N* = 1), and SHH (*N* = 1, with p-53 mutation). Of note, both of these group 3 patients had anaplastic histology and MYC amplification on original pathology.

In total, 17 patients progressed on initial therapy or relapsed (Table 2). These included tumors with anaplastic histology (*N* = 4), classical histology (*N* = 10), and desmoplastic-nodular (*N* = 3).

**Table 2:** Patient outcomes

	**Total** *N* = 30
Relapse/progression	17
SHH	3/14 (21%)
Group 3	5/7 (71%)
Group 4	6/6 (100%)
Unknown	3/3 (100%)
Current status	
Alive, without disease	17
Alive, progressive disease	2
Deceased	11
Late effects*	
Neurocognitive delay	9/19 (47%)
Hearing loss	16/19 (84%)
Endocrinopathy	8/19 (42%)

^*^Within survivors (*N* = 19)

**Fig. 2:**
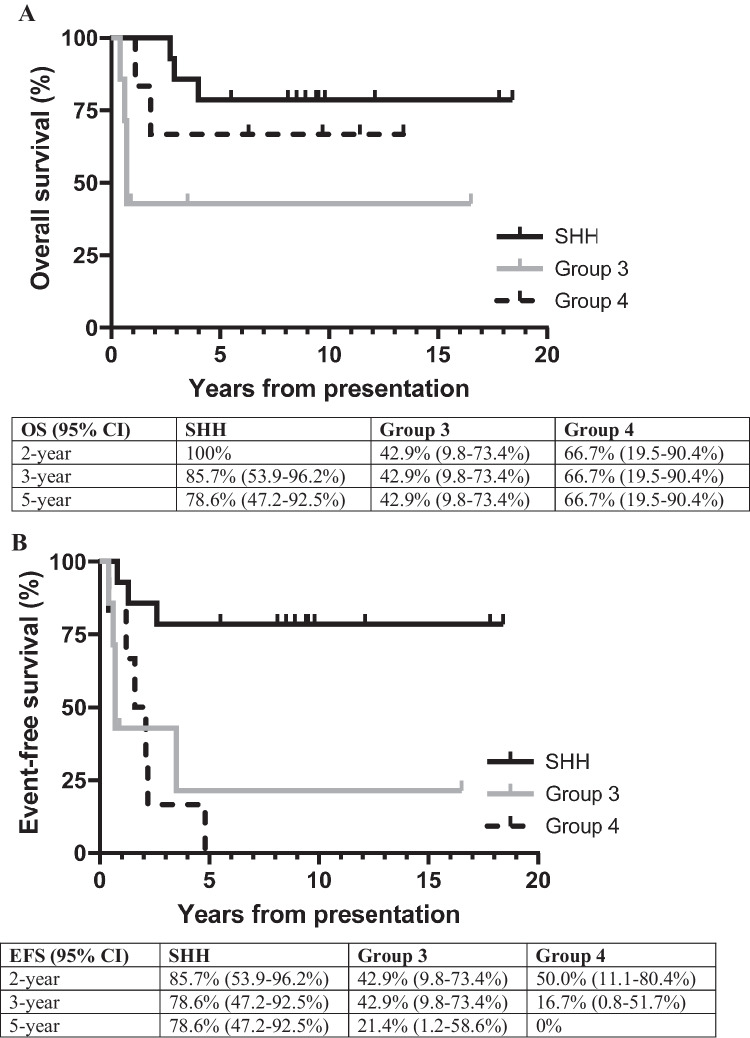
**A** Overall survival by medulloblastoma subgroup. **B** Event free survival by medulloblastoma subgroup

Within the 25 patients who achieved a CR after initial therapy, relapse occurred in 12 patients. OS by subgroup is presented in Fig. 2A and EFS by subgroup is presented in Fig. 2B. Of those patients whose disease relapsed or had progressive disease, medulloblastoma subgroup was group 4 (*N* = 6), group 3 (*N* = 5), SHH (*N* = 3), or not known (*N* = 3).

Salvage therapy at time of relapse included surgical resection (*N* = 8) followed by craniospinal RT in 7 patients (median age at radiation 6.1 [range 3.37–7.64 years]). Radiation therapy was given with 24 Gy to the craniospinal axis (CSA) and a posterior fossa boost to 56 Gy in two patients and 36 Gy to the CSA and a posterior fossa boost to 55.8 Gy in 5 patients. One patient underwent surgical resection at time of relapse and has not received further therapy. Three patients also received post-RT maintenance chemotherapy with lomustine and temozolomide. Three patients had such extensive disease at relapse that they did not receive salvage/relapse therapy and died shortly after relapse. All these patients’ tumors were identified as being group 3.

Salvage therapy was successful in 6/7 patients (group 4: *N* = 4; group 3: *N* = 1; unknown: *N* = 1), and these 6 patients are currently alive at median follow-up of 7.34 years.

### Morbidity

Summary of documented late effects within this patient population is outlined in Table 2. Of 19 patients currently alive (median follow-up 9.48 years), disease/treatment-related morbidity included endocrine hormone dysfunction (*N* = 8), hearing loss (*N* = 16), and neurocognitive abnormalities (*N* = 9).

Endocrine hormone dysfunction included panhypopituitarism (*N* = 3 patients), primary hypothyroidism (*N* = 3), precocious puberty (*N* = 1), primary gonadal failure (*N* = 1), and isolated growth delay (*N* = 1). All patients with panhypopituitarism received radiation therapy as part of salvage therapy.

Hearing loss began during treatment and was characterized as high frequency sensorineural hearing loss (HF-SNHL) in all patients. Of those with hearing loss, ten (62.5%) had SIOPE Grade III HF-SNHL. Ten of sixteen patients required the use of hearing aids at the time of last follow-up. Most patients reported the need for learning adaptations in support of hearing impairment.

No patients had visual impairment (excluding the need for corrective lenses) and none required ophthalmologic procedures.

Reported neuro-cognitive abnormalities included posterior fossa mutism, intellectual disability, memory or processing concern, or a learning disability. All nineteen survivors underwent neuropsychological testing. Three patients had reported posterior fossa mutism following upfront resection. Eight of the patients with neuro-cognitive abnormalities reported individualized education plans at school. Within the nine patients with neuro-cognitive abnormalities, five had documented intellectual disability. Of these, four patients’ neuro-psychological testing reported memory or processing abnormalities. All four of these patients had received RT. Two patients did not have a documented intellectual disability, but neuropsychological testing revealed delayed processing and memory loss. Except for an isolated learning disability, all patients with reported cognitive abnormalities underwent RT as part of salvage therapy.

There is one child who used a wheelchair for ambulation after treatment but is currently ambulating independently. One patient required a gastrostomy-tube prior to death. At the time of follow-up, there were no survivors requiring G-Tube feeds or respiratory support and no subjects with a secondary malignancy.

## Discussion

This report summarizes a single institutional cohort of 30 young patients with medulloblastoma, treated with a RT-sparing approach. To our knowledge, this is the largest cohort in the literature of single institution pediatric patients with medulloblastoma with subgrouping data treated without RT upfront. Five-year EFS and OS in this cohort of patients treated for medulloblastoma was 42.0% and 62.5%, respectively This is consistent with the reported 5-year EFS and OS (46% and 62%, respectively) of 92 children with medulloblastoma treated on the Head Start III study published in 2020 [16]. Unlike the current study, previous Head Start III publications did not include medulloblastoma molecular subgrouping.

Overall, within patients treated for medulloblastoma with RT sparing protocols, the literature is limited with regard to understanding how molecular subgrouping impact outcomes. Our results suggest that a RT sparing therapeutic approach is effective for those patients with SHH subgroup of medulloblastoma. This finding is concordant with the CCG 99703 data, although it is important to note that subgrouping was not completed on all subjects in that study and 17% of children with medulloblastoma on the CCG 99703 therapy received adjuvant RT at diagnosis. To this point, in this study, both overall survival and EFS clearly differed between medulloblastoma subgroups. In fact, OS was double in those patients with SHH when compared to group 3. In a prior Canadian collaboration, subgroup was also identified as important predictor of progression and of survivor, although within this cohort, both SHH and group 3 patients demonstrated worse overall survival when compared to group 4. Of note, this cohort included older patients (age 3 months–16.8 years) including many who received radiation therapy as part of their therapy [20]. Three- and 5-year EFS were poor within both groups 3 and 4 in the current study with noted worsening in those patients with group 4 medulloblastoma. Similar to these data, within one cohort of 29 young patients treated with chemotherapy and focal RT for medulloblastoma in Argentina, PFS was improved in tumors with SHH subtypes compared to groups 3 and 4 [17]. While the total patients were similar, this cohort had proportionately fewer group 4 patients (*N* = 2) than the data in the current study. In that study, the 5-year PFS for groups 3 and 4 patients was 0.50 (95% CI: 0.25–1); however, patients received focal RT upfront with the expected cognitive sequelae; in addition, salvage at relapse is problematic with this approach. Furthermore, while follow-up was a median of 6.8 years, neurocognitive data was only available in 11, demonstrating low average to mild intellectual disability range in the Wechsler Intelligence Scale, with a median IQ at last assessment of 73.5 (range, 47–93). This report lacks additional long-term follow-up data to assess late effects including endocrine, hearing, and vision following this treatment approach.

Within our cohort, all patients with group 4 medulloblastoma relapsed or progressed by 5 years from diagnosis. Of the six group 4 patients, four patients were salvaged with CSA RT and are now long-term survivors. It is difficult to make comparisons to other data as there are so few patients with group 4 medulloblastoma published within the RT-sparing/RT delaying literature. Within 53 patients treated as per a similar chemotherapy regimen CCG 99703, group 4 medulloblastoma did poorly when compared to the other subgroups [7]. Specifically, 5-year OS for the group 4 patients treated as per the CCG 99703 study was 33% when compared to 90% in SHH subgroup. In contrast, our data demonstrated 5-year OS for group 4 medulloblastoma of 66.7% compared to 78.6% in SHH. We suspect that the improved 5-year OS in the group 4 medulloblastoma subgroup in our data represents that group 4 medulloblastoma is salvageable with resection + CSI at the time of relapse. Thus, this is a reasonable treatment approach for young patients with group 4 medulloblastoma, so as to delay RT and help to preserve neurocognitive function.

Like group 4 medulloblastoma, 5-year PFS within the group 3 subgroup was significantly worse than in the SHH subgroup. This finding is comparable with other data in this age group [7, 17]. Unlike group 4 medulloblastoma, within the group 3 medulloblastoma patients in this study, the salvageability was poor at the time of relapse. Overall survival at 5 years was 42.9%, significantly lower than SHH or group 4. More than two-thirds of group 3 patients relapsed, of which only one patient was salvaged with gross total resection and CSA RT. That patient had a localized relapse, whereas the other 4 patients within that subgroup had disseminated disease relapse in addition to MYC amplification on molecular testing. Histology may also have contributed to these poor outcomes, with two of these patients also having anaplastic histology. More effective radiation sparing strategies are needed for group 3 medulloblastoma to reduce disseminated failures in the MYC-amplified patients in particular.

Forty percent of patients in this cohort received high-dose systemic methotrexate as part of their chemotherapy. In the Head Start I and II trials, methotrexate was reserved for disseminated disease [4] and in Head Start III, methotrexate was included as part of therapy during induction in three out of 5 cycles [16]. Our institutional standard of care was changed to include methotrexate for all patients with medulloblastoma (as per the Head Start III trial) based on published data from the early Head Start trials demonstrating improved EFS for patients with dissemination when methotrexate was added [14]. Within the current study, 75% of patients who received methotrexate had a complete response to upfront therapy, which is consistent with reports from the Head Start trials where 81% of patients treated with methotrexate had a complete response [14]. Of note, that data included 21 patients. Within group 3 and group 4 patients, the rate of relapse in the current study in those who received methotrexate was 1/3 less than within those that did not receive methotrexate. Although small numbers and thus should be interpreted with caution, these data suggest that methotrexate is important within these subgroups.

Many patients within this cohort experienced treatment-related late effects, with the most common late effect being hearing loss. Sensorineural hearing loss was seen in 94% of patients, with 62.5% having grade III hearing loss. This is consistent with prior late-effects data for patients treated on the Head Start I and II studies where grade III hearing loss was seen in 67% of survivors [24]. This is likely due to platinum-based chemotherapy included in all of the Head Start protocols, which may have been further exacerbated by RT effects in those who received salvage therapy. Similarly, endocrinopathies were noted sporadically within survivors in this cohort, although at higher rates than prior publications that included patients who did not ever receive RT [25]. Of note, all but one patient in the current study with endocrine dysfunction received salvage RT.

The impact of treatment can be moderated by several factors, including age, disease, and type of treatment with children at a young age being at greatest risk of neurocognitive sequelae. Due to this, neurocognitive testing is tailored to the individual patient and made the current data challenging to draw strong conclusions from. Neurocognitive late effects were reported in a small proportion of patients within this cohort; in the majority, this was limited to those who required craniospinal radiation salvage therapy; however, this data must be interpreted within the limitations of clinician reporting. In contrast, within the Argentinian cohort of young patients with medulloblastoma treated with chemotherapy ± focal RT, most survivors had borderline to mild intellectual disability [17]. It should be noted that in the study in Argentina, only 11 patients had neurocognitive testing available. It is possible that testing was biased towards those patients with neuro-cognitive symptoms, but also testing was potentially limited by profound dysfunction or by economic factors. Within those patients treated as per CCG 99703, mean FSIQ for 24 survivors was 91.6 (range 52–119) [7], slightly lower than within our cohort, it should be noted that 32% of those patients received either focal or CSA RT either adjuvant to upfront chemotherapy or at relapse; the majority of those who were impaired had received RT.

### Strengths and limitations

The results from this cohort must be interpreted within their limitations. The main limitation of this data set is its retrospective nature, and the clinical data is limited by what information was available in the patient record. Despite this, all subjects had complete data available for analysis. Furthermore, these data represent care at a single institution with consistent providers for the entirety of this dataset. As such, there was limited variability in terms of management. As well, this cohort’s OS and EFS were consistent with prior published data in RT-sparing approaches for medulloblastoma in young children. While the relatively long follow-up time is a strength of this data and allows for clear description of patients’ morbidity, in many instances, baseline testing (including hearing, neurocognitive measures, and endocrine function) was not available with which to elicit change from baseline. As such, we cannot be sure that all the described morbidity in this paper are due to treatment received.

Despite some limitations, an important strength of this data is that it is the largest published cohort of patients treated upfront with a RT sparing approach for medulloblastoma from a single institution with molecular subgrouping data. Furthermore, no patients were lost to follow-up and median follow-up was more than 9 years, allowing for extensive assessment of clinical outcomes and treatment-related morbidity.

## Conclusions

Radiation therapy sparing treatment approach for young patients with medulloblastoma resulted in a durable cure in most patients with SHH subgroup. In those patients with groups 3 and 4 medulloblastoma, relapse rates were high; however, most group 4 patients were salvaged with gross total resection followed by CSA RT. These data suggest that improved therapeutic strategies are sorely needed for patients with group 3 medulloblastoma and more intensive therapy may be necessary upfront to achieve a durable cure for both groups 3 and 4 tumors.

## Data Availability

Dataset is available upon request from the corresponding author.
